# Supplementary material for: Adaptation to acidic conditions that mimic the tumor microenvironment, downregulates miR-193b-3p, and induces EMT via TGFβ2 in A549 cells
Source: PLoS One. 2025 Feb 24;20(2):e0318811. doi: 10.1371/journal.pone.0318811 (PMC12140115; doi:10.1371/journal.pone.0318811)
Supplement: S1 Table — (PDF) [file pone.0318811.s001.pdf]

S1 Table. primary antibodies in this study.

primary antibodies for western blot

Gene name	Company (Catalog #)	Dilution rate
GAPDH	SANTA CRUZ BIOTECHNOLOGY (sc-47724)	1:2000
Vimentin	SANTA CRUZ BIOTECHNOLOGY (sc-373717)	1:2000
E-Cadherin	Cell Signaling Technology (#3195S)	1:1000
N-Cadherin	Cell Signaling Technology (#13116S)	1:1000
