# Supplementary material for: Adaptation to acidic conditions that mimic the tumor microenvironment, downregulates miR-193b-3p, and induces EMT via TGFβ2 in A549 cells
Source: PLoS One. 2025 Feb 24;20(2):e0318811. doi: 10.1371/journal.pone.0318811 (PMC12140115; doi:10.1371/journal.pone.0318811)
Supplement: S2 Table — (PDF) [file pone.0318811.s002.PDF]

S2 Table. Sequences of gene-specific primers for RT-PCR and primer ID in this study.

Gene-specific primers

**Gene name**	**Forward primer (5' – 3')**	**Reverse primer (5' – 3')**
TGFB1	TACCTGAACCCGTGTTGCTCTC	GTTGCTGAGGTATCGCCAGGAA
TGFB2	AAGAAGCGTGCTTTGGATGCGG	ATGCTCCAGCACAGAAGTTGGC
GAPDH	GTCTCCTCTGACTTCAACAGCG	ACCACCCTGTTGCTGTAGCCAA

Primer ID

**microRNA name**	**Company**	**ID**
hsa-miR-193b-3p	Applied biosystems	002367
U6	Applied biosystems	001973
