# Supplementary material for: Adaptation to acidic conditions that mimic the tumor microenvironment, downregulates miR-193b-3p, and induces EMT via TGFβ2 in A549 cells
Source: PLoS One. 2025 Feb 24;20(2):e0318811. doi: 10.1371/journal.pone.0318811 (PMC12140115; doi:10.1371/journal.pone.0318811)
Supplement: S1 Fig — (PDF) [file pone.0318811.s003.pdf]

supplementary Fig 1

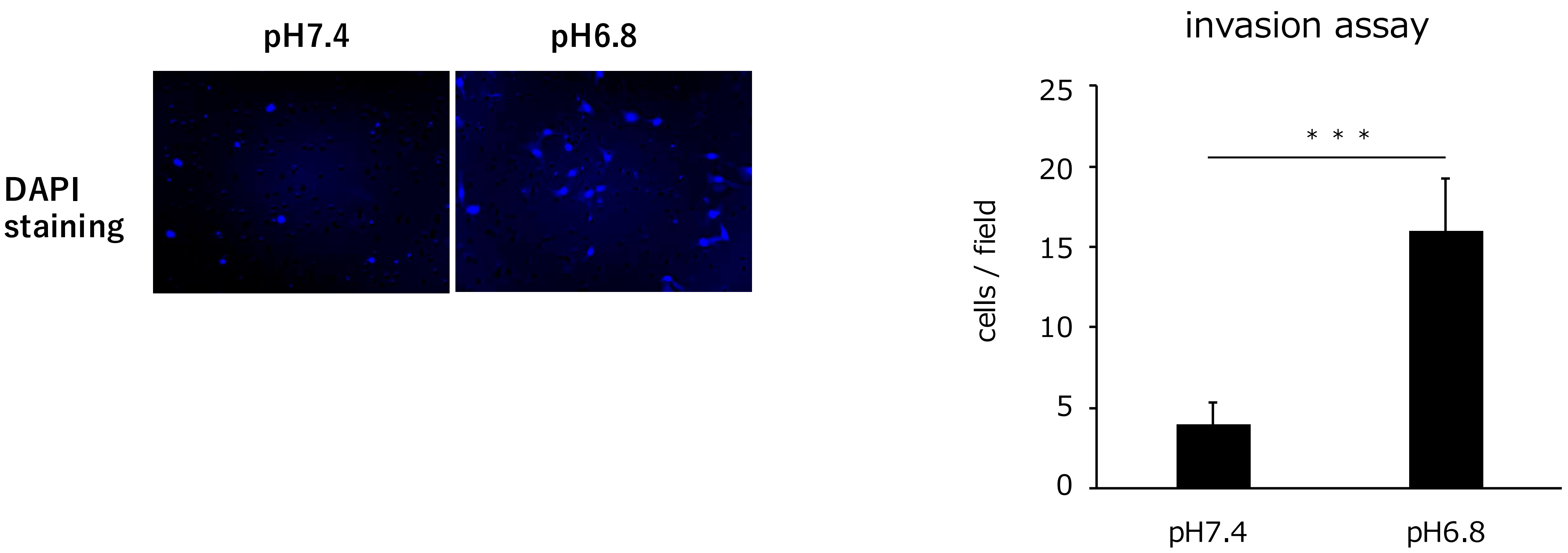

Cells were seeded into Matrigel invasion chambers (24-well Plate, 8.0 Micron, CORNING) at a density of 2 × 10^4 cells/well. The cells were adapted to either pH 7.4 or pH 6.8 conditions for 8 weeks prior to the experiment. The chambers were incubated at 37° C for 24 hours. After incubation, the Matrigel on the membrane was removed using a cotton swab. The cells were then fixed with 4% paraformaldehyde for 2 minutes and permeabilized with 0.25% Triton-X 100. Nuclei were stained with DAPI (1:1000 dilution; PureBlu DAPI, BIO-RAD) for 1 minute. The samples were washed three times with PBS, and the membranes were observed under a microscope at 200 × magnification. The number of cells per field of view was counted.

Note: For the pH 7.4 condition, n=8; for the pH 6.8 condition, n=9. Statistical analysis was performed using an unpaired t-test.

*** $P < 0.001$ indicates a significant difference compared to the pH 7.4 condition.
