# Supplementary material for: Adaptation to acidic conditions that mimic the tumor microenvironment, downregulates miR-193b-3p, and induces EMT via TGFβ2 in A549 cells
Source: PLoS One. 2025 Feb 24;20(2):e0318811. doi: 10.1371/journal.pone.0318811 (PMC12140115; doi:10.1371/journal.pone.0318811)

Supplymentary2_Fig

Figure 1C

The area surrounded by a line is used as a figure.
The marker positions are indicated by attaching an image taken with visible light next to the chemiluminescence detection image.

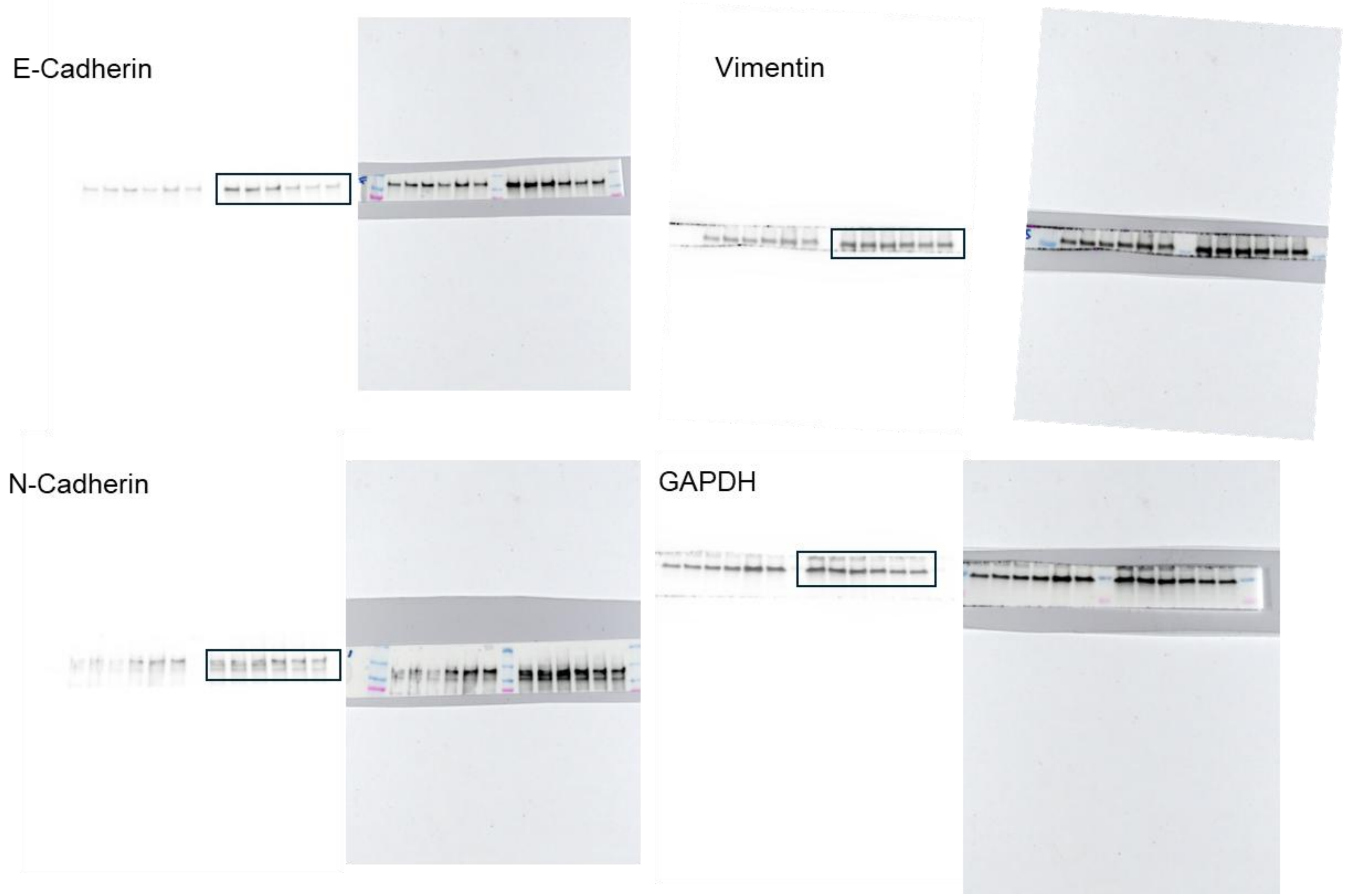

Figure 2A-1

The area surrounded by a line is used as a figure.
The marker positions are indicated by attaching an image taken with visible light next to the chemiluminescence detection image.

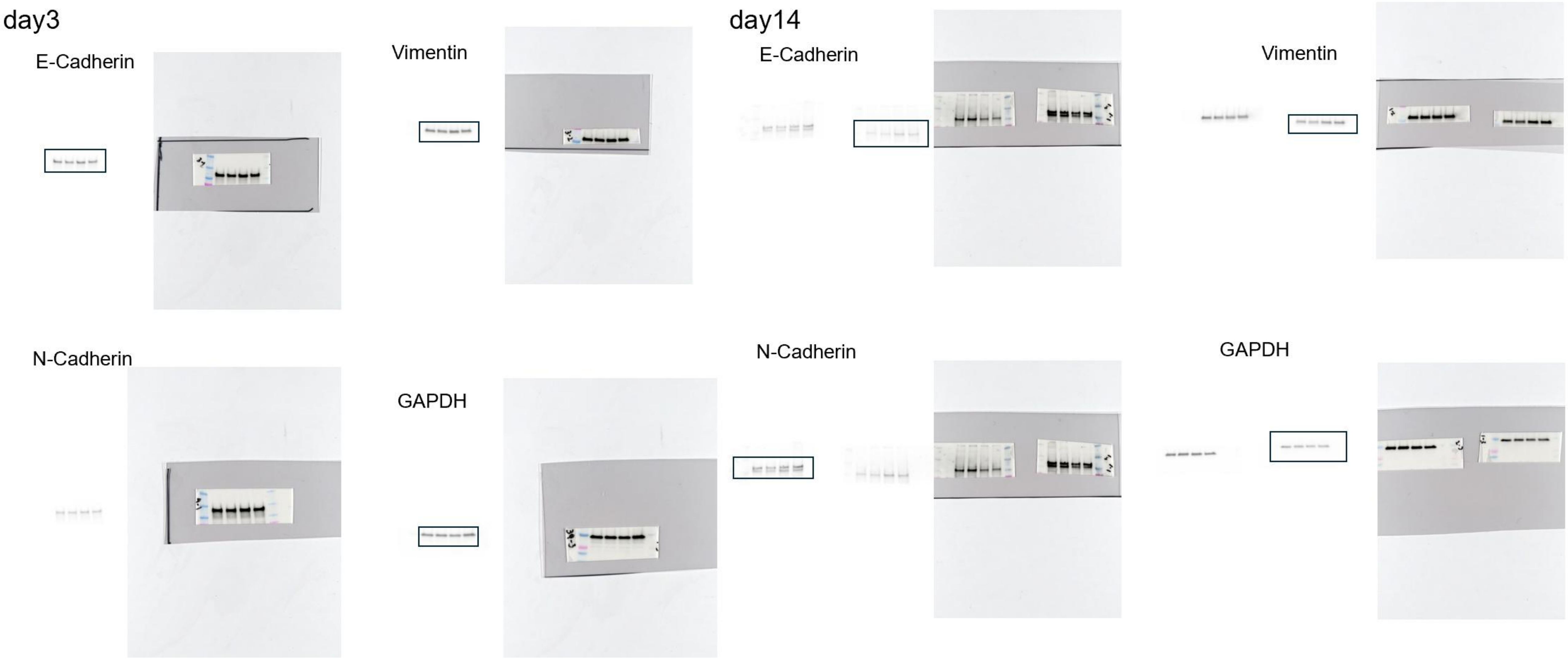

Figure 2A-2

The area surrounded by a line is used as a figure.
The marker positions are indicated by attaching an image taken with visible light next to the chemiluminescence detection image.

day28

E-Cadherin

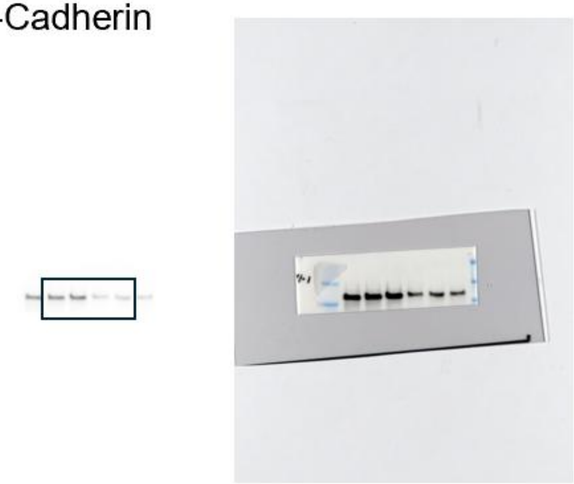

Vimentin

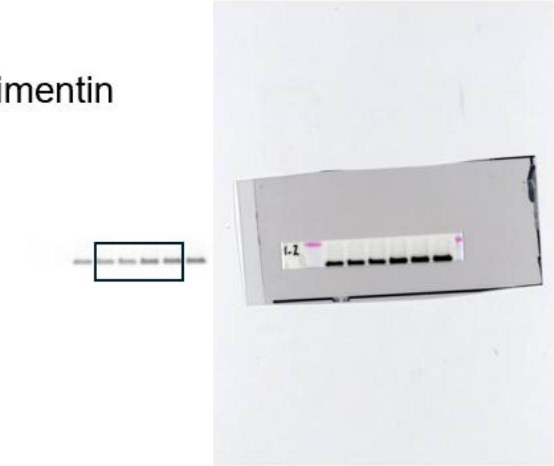

N-Cadherin

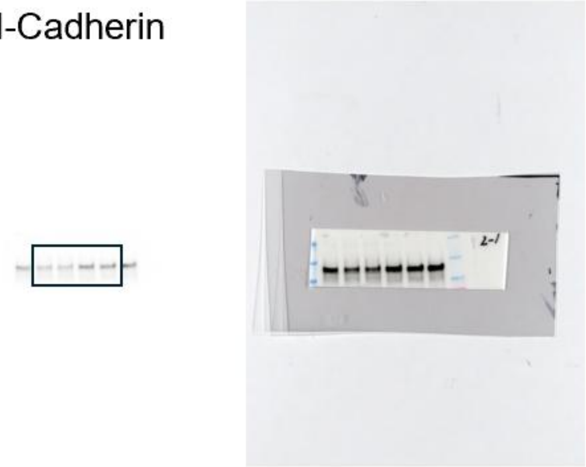

GAPDH

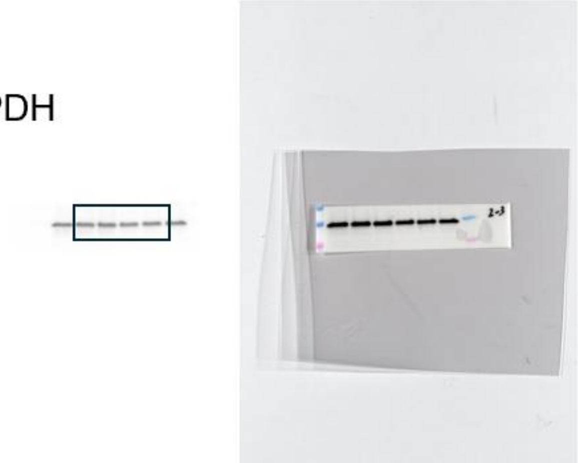

Figure 2C

The area surrounded by a line is used as a figure.
The marker positions are indicated by attaching an image taken with visible light next to the chemiluminescence detection image.

E-Cadherin

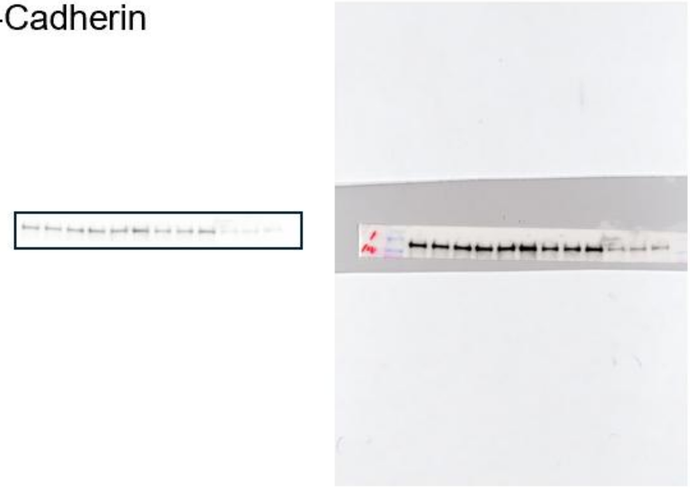

Vimentin

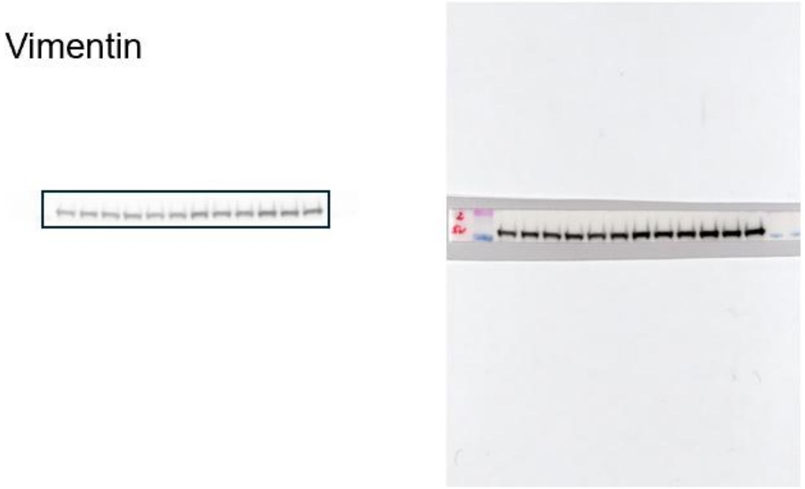

N-Cadherin

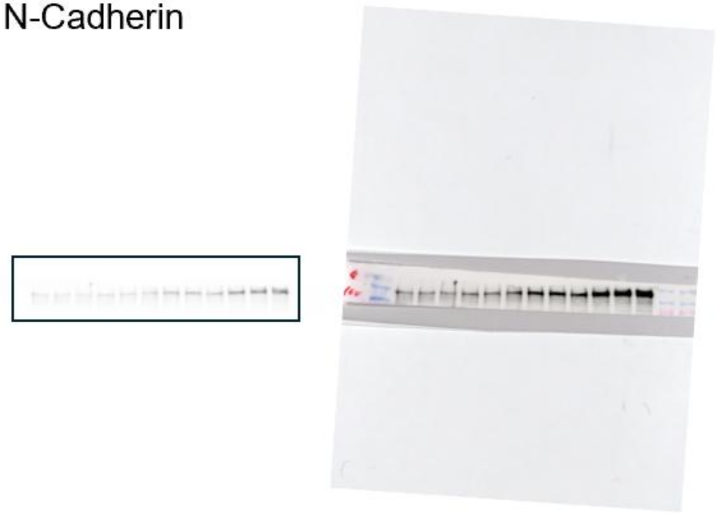

GAPDH

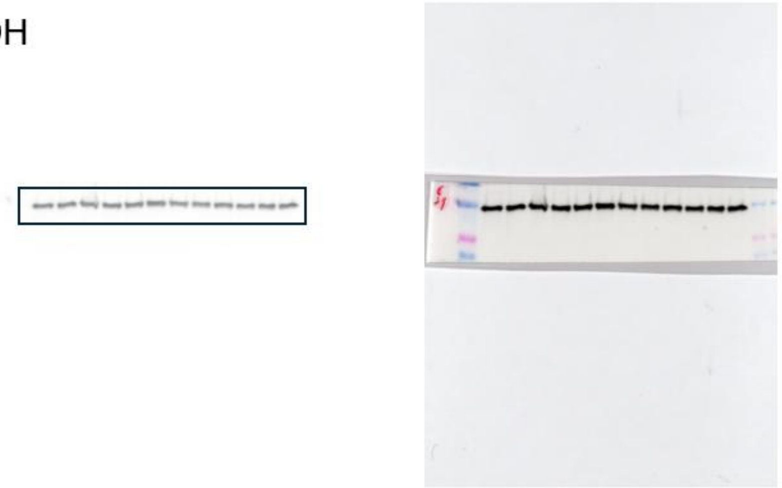

Figure 2D

The area surrounded by a line is used as a figure.
The marker positions are indicated by attaching
an image taken with visible light next to the
chemiluminescence detection image.

E-Cadherin

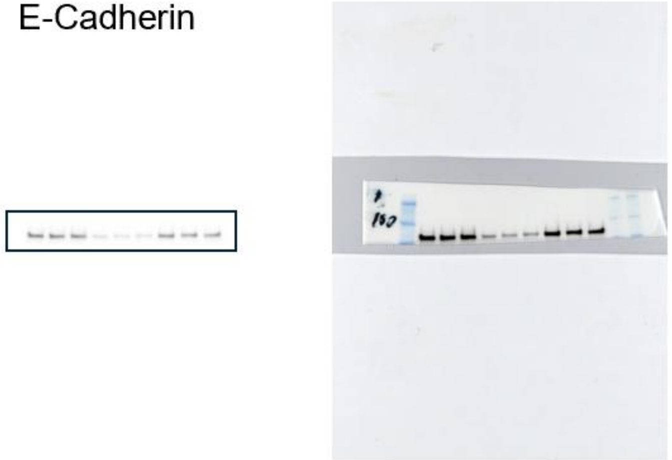

Vimentin

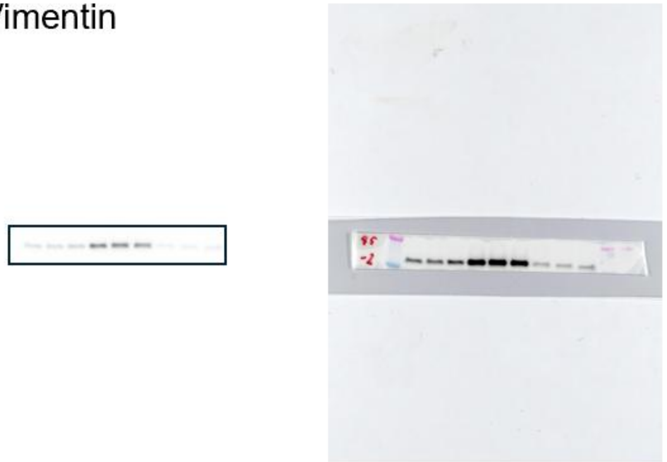

N-Cadherin

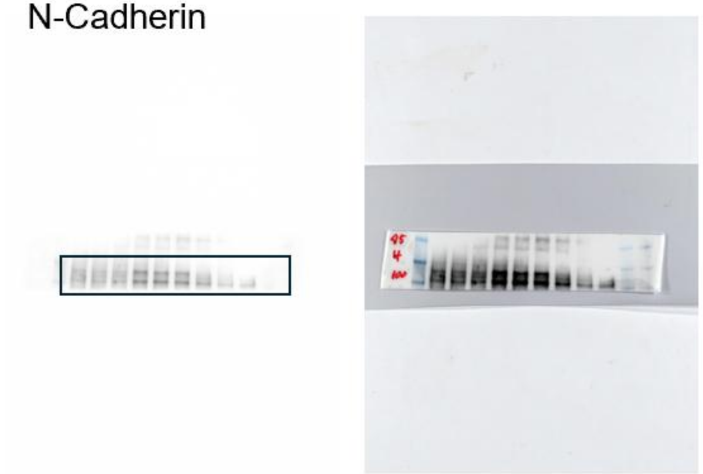

GAPDH

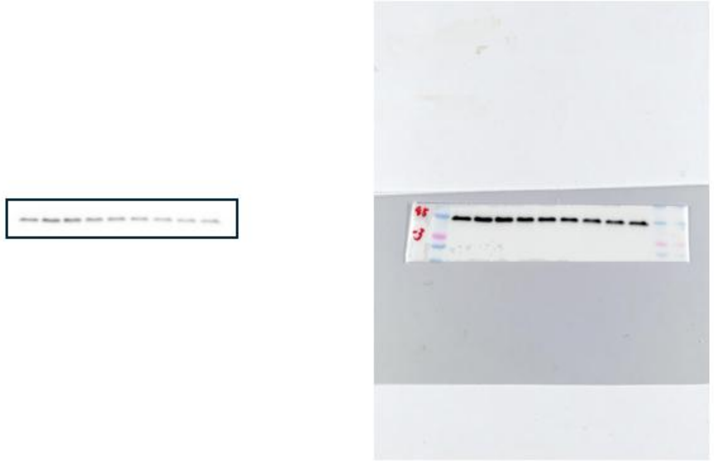

Figure 4C

The area surrounded by a line is used as a figure.
The marker positions are indicated by attaching an image taken with visible light next to the chemiluminescence detection image.

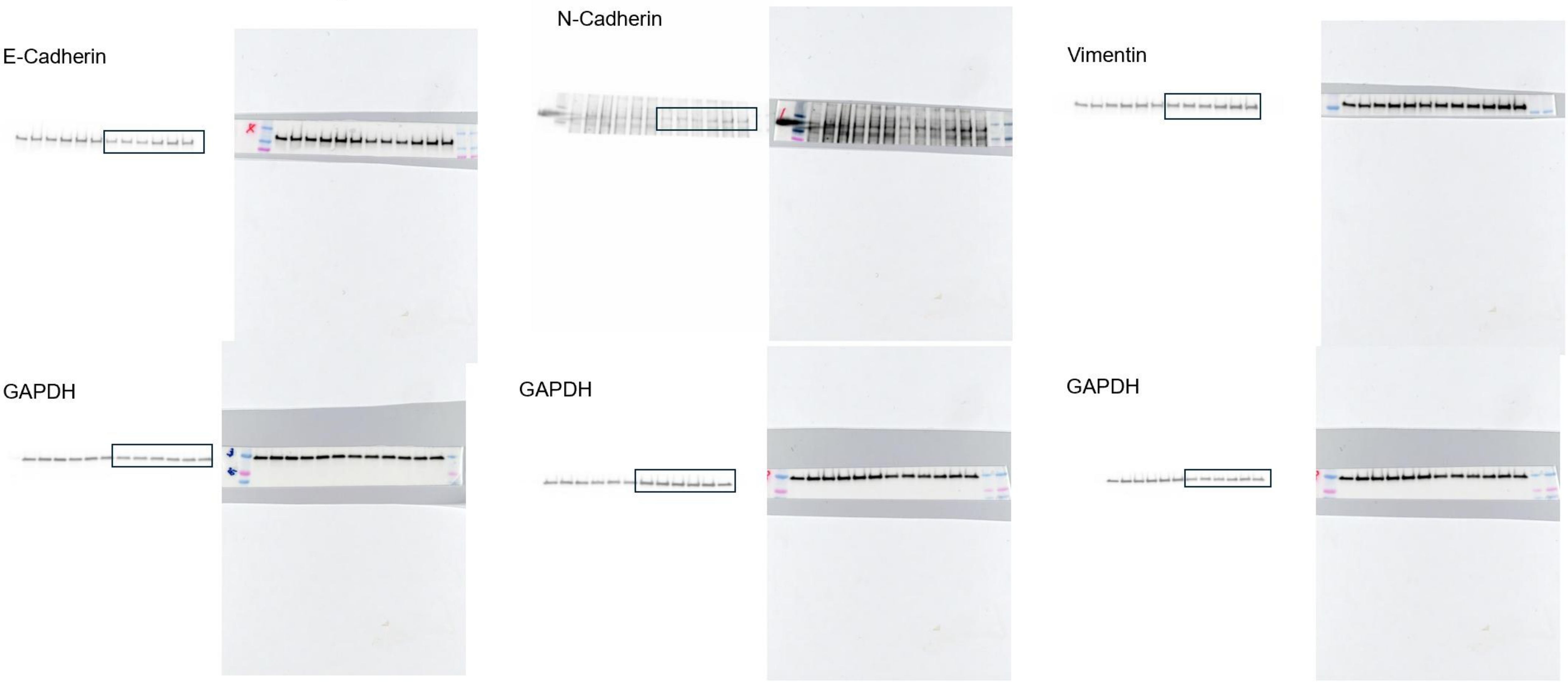

Figure 4D

The area surrounded by a line is used as a figure.
The marker positions are indicated by attaching
an image taken with visible light next to the
chemiluminescence detection image.

E-Cadherin

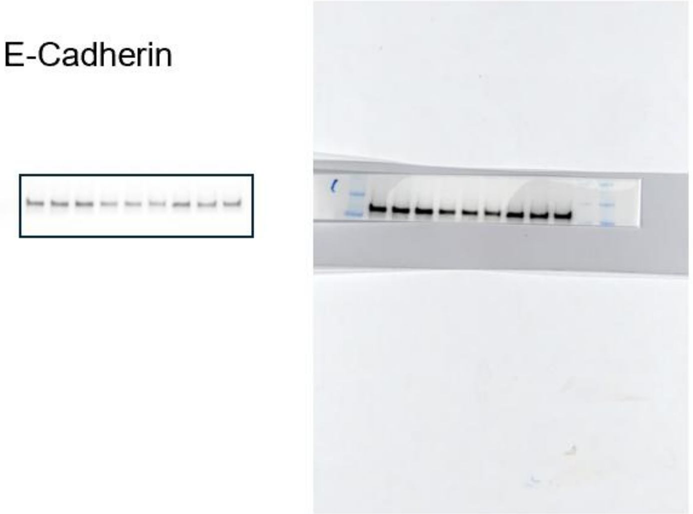

Vimentin

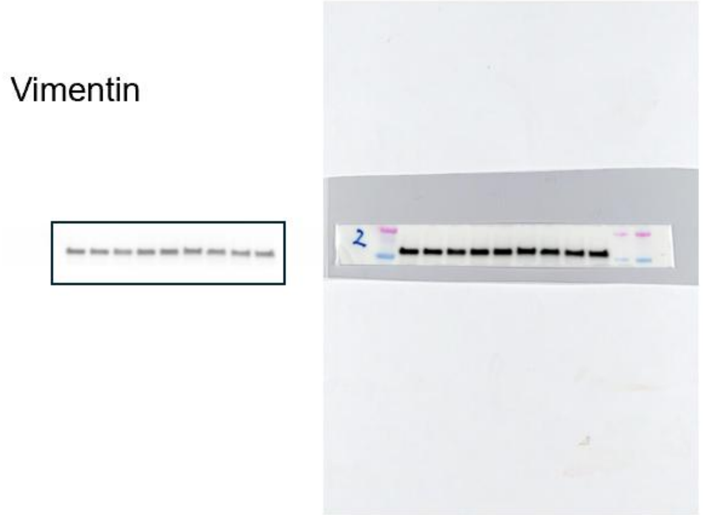

N-Cadherin

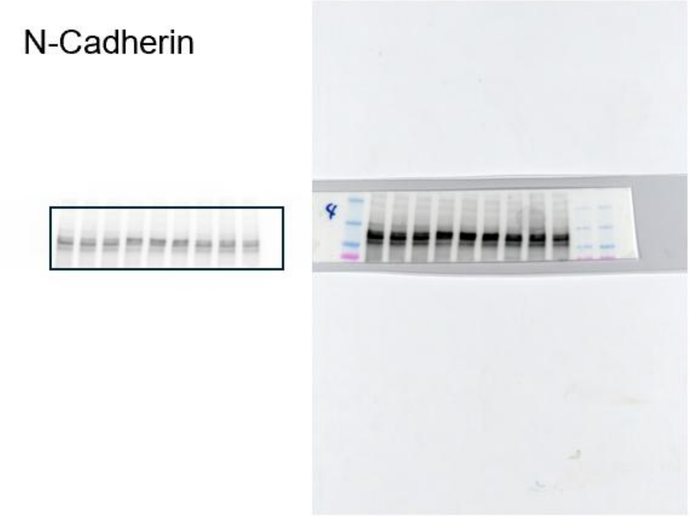

GAPDH

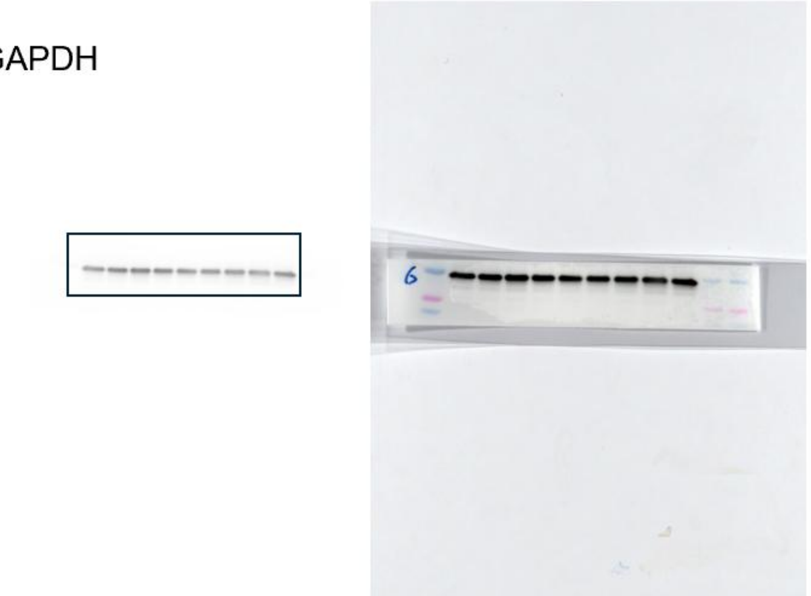

Figure 4G

The area surrounded by a line is used as a figure.
The marker positions are indicated by attaching
an image taken with visible light next to the
chemiluminescence detection image.

E-Cadherin

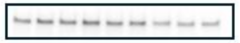

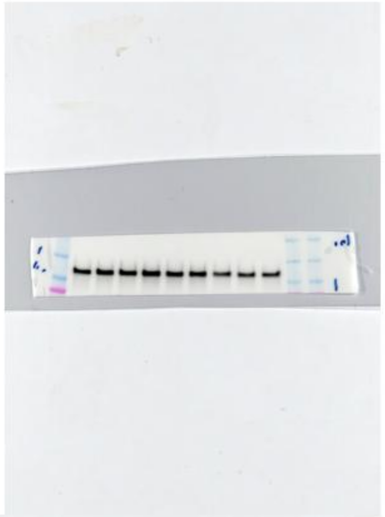

Vimentin

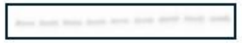

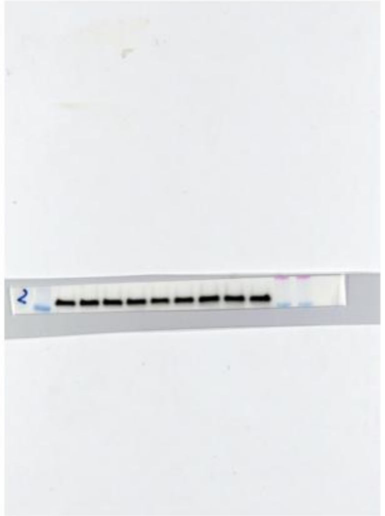

N-Cadherin

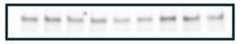

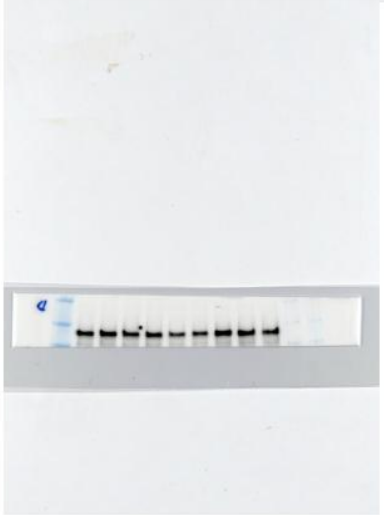

GAPDH

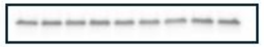

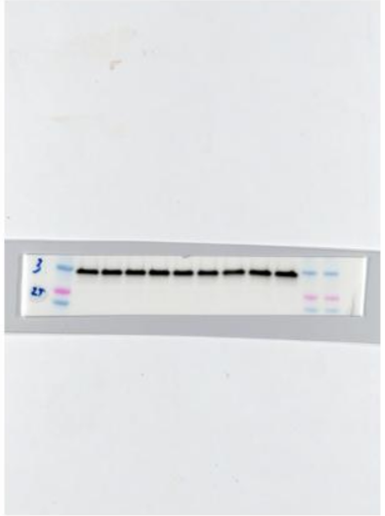

Supplement: S2 Fig — (PDF) [file pone.0318811.s004.pdf]
